# Oestrogenic Regulation of Mitochondrial Dynamics

**DOI:** 10.3390/ijms23031118

**Published:** 2022-01-20

**Authors:** Siavash Beikoghli Kalkhoran, Georgios Kararigas

**Affiliations:** Department of Physiology, Faculty of Medicine, University of Iceland, 101 Reykjavik, Iceland; kalkhoran@hi.is

**Keywords:** 17β-oestradiol, biological sex, cardiovascular, heart–brain axis, neuronal

## Abstract

Biological sex influences disease development and progression. The steroid hormone 17β-oestradiol (E2), along with its receptors, is expected to play a major role in the manifestation of sex differences. E2 exerts pleiotropic effects in a system-specific manner. Mitochondria are one of the central targets of E2, and their biogenesis and respiration are known to be modulated by E2. More recently, it has become apparent that E2 also regulates mitochondrial fusion–fission dynamics, thereby affecting cellular metabolism. The aim of this article is to discuss the regulatory pathways by which E2 orchestrates the activity of several components of mitochondrial dynamics in the cardiovascular and nervous systems in health and disease. We conclude that E2 regulates mitochondrial dynamics to maintain the mitochondrial network promoting mitochondrial fusion and attenuating mitochondrial fission in both the cardiovascular and nervous systems.

## 1. Introduction

Biological sex plays a major role in disease development and treatment responses [1,2,3,4,5,6]. This is true for a wide range of diseases, including cardiovascular diseases, as well as nervous system-related diseases (neuropsychiatric, neurological and neurodegenerative disorders) [7,8,9,10,11,12,13,14]. In addition to sex chromosomes and (epi)genetic factors [1,7,8,15], steroid hormones are expected to be one of the main factors that drive differences between the sexes. In particular, 17β-oestradiol (E2) and its receptors (ER) are thought to play a major role [16,17,18,19,20,21].

E2 biosynthesis primarily occurs in the gonads. Consequently, in pre-menopausal women, E2 is produced mainly by the ovaries. However, production of E2 may continue post menopause, as there are extra-ovarian sources of E2. These include the adipose, breast and adrenal tissues, bone, heart, brain and skin, where aromatase can be produced [22]. In addition, the testes and prostate are production sites of E2 through the local conversion of androgenic precursors by the aromatase enzyme. Following production, E2 is released into the bloodstream, where it diffuses across the cellular plasma membrane binding to different intracellular receptors, including ERα, ERβ and G protein-coupled oestrogen receptor (GPER), to elicit its effects. A wide range of E2/ER effects have been studied in the cardiovascular and nervous systems [23,24,25,26,27,28,29,30,31,32,33,34,35,36,37,38,39,40], which can be sex-dependent [18,22,41,42,43,44,45,46] and demonstrate the modulatory role of E2 in these systems.

E2 induces its diverse effects by modulating numerous signalling pathways. Mitochondria are one of the essential cellular organelles for the induction of E2 effects [47]. Regulation of mitochondrial metabolism [24,48], cell death [49,50] and quality control [51,52] are a few examples by which E2 regulates cellular homeostasis. In this article, we discuss the mechanisms of E2 actions and its effects on mitochondrial function. We then specifically focus on the interplay of E2 with mitochondrial function and dynamics in the cardiovascular and neurological systems and explore the involvement of E2 in mitochondrial dynamics in the heart–brain axis (HBA).

## 2. E2 Regulation of Mitochondrial Function and Turnover

### 2.1. E2 Effects on Mitochondrial Respiration

Mitochondria are crucial for cellular metabolism, where most of the cellular ATP is produced [53]. Mitochondrial catabolic processes, such as glycolysis and β-oxidation, either directly produce reducing agents, including NADH and FADH_2_, or indirectly by supplementing the mitochondrial tricarboxylic acid cycle with pyruvate or acetyl-CoA. The reducing agents are then used to feed electrons to complexes I and II of the electron transport chain for oxidative phosphorylation. The electron transfer results in outward movements of protons across the inner mitochondrial membrane (IMM) through different respiratory complexes. This results in the formation of an electrical gradient across the mitochondrial membrane that is necessary for ATP synthesis [54].

**Figure 1:**
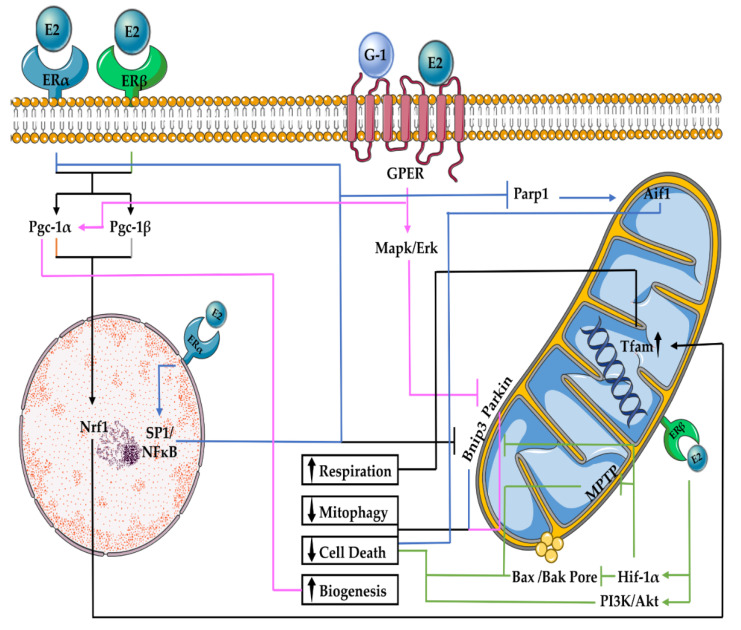
E2 regulation of mitochondrial function and turnover. Several signalling proteins contribute to the effects of E2 on mitochondrial respiration, biogenesis, mitophagy and cell death. Abbreviations: Aif1: Apoptotic-inducing factor 1, E2: 17β-Oestradiol, ERα: oestrogen receptor α, ERβ: oestrogen receptor β, Erk: extracellular signal-regulated kinase, Mapk: mitogen-activated-protein-kinase, MPTP: mitochondria permeability transition pore, Nfκb: Nuclear factor kappa-light-chain-enhancer of activated B cells, Nrf1: nuclear receptor factor, Pgc-1α: proliferator-activated receptor-γ coactivator 1-α, Pgc-1β: proliferator-activated receptor-γ coactivator 1-β, PI3K: phosphatidylinositol-3-OH kinase, Sp1: stimulating protein-1 and Tfam: mitochondrial transcription factor A.

E2 regulation of mitochondrial function often requires its interaction with its receptors (Figure 1). Apart from GPER, which is localised in the endoplasmic reticulum (ERT) or plasma membrane, other ERs are additionally localised on the surface of mitochondria and are expressed in a tissue-dependent manner [55]. ERα and ERβ have been found in cardiomyocytes and neurons [56,57]. However, the presence of ERβ in cardiomyocytes, particularly in mitochondria, has been disputed [58]. Nevertheless, ERβ seems to be the main ER present in mitochondria, as demonstrated by immunohistochemistry, immunocytochemistry and immunoblots, using a large panel of antibodies [26]. In addition, studies indicating its functional activity there have been reported [24].

The E2–ER interaction either follows a direct regulation of mitochondrial DNA (mtDNA) via E2 response element sites or indirect activation of proteins, such as nuclear receptor factor 2 (Nrf2), which binds to the peroxisome proliferator-activated receptor-γ coactivator (Pgc) 1α transcription factor [59]. Pgc-1α then interacts with nuclear receptor factor 1 (Nrf1), which promotes mitochondrial transcription factor A (Tfam) expression, and regulates mitochondrial protein synthesis and respiration [60,61,62]. In line with this notion, E2 uptake in male athletes resulted in higher levels of skeletal muscle PGC-1α and medium-chain acyl-CoA dehydrogenase (β-oxidation substrate), as well as greater lipid oxidation compared with the placebo [60]. Similarly, in cerebrovascular tissues of ovariectomised (OVX) rats, E2 treatment enhanced mitochondrial respiration via increase of mitochondrial cytochrome C and complex IV through the function of Nrf2 [56]. Together, these studies reveal the significance of E2 in promoting the performance of mitochondrial respiration.

### 2.2. E2 Effects on Mitophagy

In order to work in an optimal manner, the mitochondrial network eliminates dysfunctional mitochondria from the cell through the mechanism of mitophagy, while it generates new mitochondria via the process of biogenesis (discussed later). Removal of mitochondria involves receptor-mediated mitophagy either via Bnip3/Nix or Fundc1 or ubiquitin-mediated mitophagy via Parkin and Pink1. In the first type, Bnip3 or Fundc1 travel to the surface of mitochondria [63]. The phosphorylation of Bnip3 at its serine 212 site and its subsequent dimerization at the outer mitochondrial membrane (OMM) are essential for its mitophagic activity [64]. Comparatively, under basal conditions, Src and CK2 kinases phosphorylate Fundc1 at its tyrosine 18 and serine 13 sites, respectively, and allow its association with mitochondrial fusion protein optic atrophy 1 (Opa1) via its lysine 70 site [63,65,66]. Under stress conditions, such as hypoxia, dephosphorylation of Fundc1 via Pgams5 phosphatase and its dissociation from Opa1 allow Fundc1 translocation to the OMM to instigate mitochondrial fragmentation [66]. Both Bnip3 and Fundc1 act as a receptor for microtubule-associated proteins 1A/1B light chain 3B (LC3) or GABAPAR proteins that ultimately facilitate mitophagosome formation [63]. In Parkin-Pink1-dependent mitophagy, the loss of mitochondrial membrane potential triggers Pink1-induced phosphorylation of ubiquitin. The phosphorylation of ubiquitin allows Parkin translocation to the OMM. Parkin phosphorylation at its serine 65 by Pink1 then allows its conformational changes and subsequent ubiquitylation of several proteins on the OMM before mitophagosome formation [67].

E2 interacts with components of both receptor- and ubiquitin-mediated mitophagy. Under stress conditions, such as hypoxia, Bnip3 interferes with the activity of the anti-apoptotic Bcl2 and Rheb proteins, which activate a mitophagic inhibitor known as mammalian target of rapamycin (mTOR) [68]. E2/ERα signalling has been observed to normalise mitophagy and rescue cardiac H9C2 cells overexpressing Bnip3, by actively competing with Bnip3 to bind to Bcl2 and Rheb. E2/ERα was also postulated to reduce the intracellular levels of Bnip3 via Sp1 and nuclear factor kappa-light-chain-enhancer of activated B cells (NfκB) transcription factors [69]. Similarly, in amyloid-beta (Aβ)-induced cytotoxicity, E2 treatment lowers Bnip3 levels in primary cerebrocortical neuronal cells [70]. E2 can also rely on ERβ to reduce Bnip3 through the action of hypoxia-inducing factor 1 α (Hif1-α) [71]. Relatively, in E2-treated OVX mice subjected to ischaemia-reperfusion injury (IRI), GPER activation reduced Parkin translocation to the OMM, thereby decreasing mitophagy. Inhibition of Pink-Parkin-related mitophagy, in line with the induction of Mapk/Erk, has been observed to reduce cardiac infarct size, independent of the PI3k/Akt pathway in E2-treated OVX mice [72]. Collectively, E2 modulates diverse signalling pathways to regulate mitophagy based on cellular homeostasis.

### 2.3. E2 Effects on Mitochondrial Biogenesis

Proteins that actively participate in the regulation of mitochondrial metabolism, including Pgc-1α, Pgc-1β, Nrf1, Nrf2, Tfam and peroxisome proliferator-activated receptor-γ (PPARγ), act cooperatively to regulate mitochondrial biogenesis [73]. E2 can have both direct and indirect effects on mitochondrial biogenesis. OVX mice exhibit a marked downregulation of Pgc-1α and Tfam. E2 treatment restored Tfam, as well as Pgc-1α levels, in part via upregulation of Sirt1 and Sirt3, which are known to deacetylate and enhance Pgc-1α activity [74]. E2 treatment in OVX rats increased Pgc-1β levels, which in turn activated Tfam and Nrf1 and improved mitochondrial respiration and biogenesis in the liver and cerebral blood vessels [75,76]. In addition, E2 treatment can increase GPER levels, while simultaneously increasing Pgc-1α levels. Interestingly, GPER agonist G-1 was able to mimic these effects (Figure 1). However, no effects on biogenesis markers were observed following ERα or ERβ agonists (PTT and DPN, respectively) treatment, thereby suggesting that the effects of E2 on the biogenesis of cardiac mitochondria are GPER-dependent [77].

### 2.4. E2 Effects on Mitochondrial-Associated Cell Death

The induction of numerous cell death pathways begins from mitochondria. After receiving cell death-associated stimuli, members of the Bcl2 family of proteins translocate to the surface of mitochondria to form a pore. This then causes mitochondrial outer membrane permeabilisation and the release of apoptotic-inducing factor 1 (Aif1), cytochrome C and SMAC proteins into the cytosol. These proteins then activate caspases and nucleases, which ultimately induce apoptosis [78]. Aif1 translocation for the induction of apoptotic-like cell death also occurs in parthanatos. Here, hyperactivation of poly(ADP-ribose) polymerase 1 (Parp1) allows Aif1 translocation to the cell nucleus triggering nuclease activation, which subsequently causes DNA fragmentation and cell death [79]. Necrotic cell death involves the formation of necrosomes via receptor-interacting protein (Rip) kinases, including Rip1 and Rip3. Necrosome formation, along with a drop in mitochondrial membrane potential, induces the opening of a small pore, known as the mitochondrial permeability transition pore (MPTP), which increases the mitochondrial permeability to cause swelling, ultimately leading to cell death [80]. The extent of necrosis differs between males and females, as female mitochondria have higher Ca^2+^ capacity and as a result, present less Ca^2+^-induced swelling and MPTP [81]. The E2 protection against MPTP opening has been observed to occur mainly through the function of ERβ [82]. In neurons and cardiomyocytes, E2 treatment activates the pro-survival PI3K/Akt pathway to prevent ischaemia-induced apoptosis [83,84]. The effect of E2 under hypoxic conditions includes the activation of Hif1-α by ERβ, which decreases apoptotic pore-forming Bax and Bak protein levels [71]. E2 treatment can also inhibit the DNA binding of NfκB in cardiomyocytes via ERα and ERβ, thereby preventing inflammation-related apoptosis [85]. Conversely, E2 can also stimulate apoptosis in the hypothalamus regulating the ratio of dopaminergic neurons [86]. Unlike the other two cell death pathways, E2 relies on the action of ERα to reduce Parp1 activation following oxidative stress [87]. Therefore, E2 exerts regulatory effects on a range of cell death pathways that affect mitochondria by utilising different receptors.

## 3. Mitochondrial Dynamics

### 3.1. Mitochondrial Fusion 

Mitochondria are active organelles that constantly change shape via fusion and fission processes, collectively known as mitochondrial dynamics, to respond to cellular demands. Several dynamin-related GTPases orchestrate mitochondrial dynamics. Neighbouring mitochondria merge their membrane and fuse using the OMM proteins Mfn1 and Mfn2, as well as the short and long isoforms of the protein Opa1 that reside in the IMM [88]. Mitochondrial fusion is essential for mtDNA repair, stability and replication [89,90], UV and starvation-induced hyperfusion [91], network distribution, cell survival and embryonic development [92]. The action of mitofusins begins by reducing the strength of the OMM lipid layer via their Heptad-Repeat 1 domain. The Heptad-Repeat 2 domain then uses GTP to bring the OMM of adjacent mitochondria together to fulfil the OMM fusion with the action of other mitofusin domains [93]. Downstream of mitofusins, mitoguardin proteins, Miga1 and Miga2, interact with MitoPLD and lead to its dimerization [94]. MitoPLD then forms phosphatidic acid phospholipids by using cardiolipin to facilitate membrane fusion [95].

The fusion of the IMM relies on the activity of the Opa1 protein. The proteolytic cleavage of Opa1 results in the formation of long Opa1 (L-Opa1) and short Opa1 (S-Opa1) isoforms [96]. Both isoforms hydrolyse GTP to create homo- and heterotypic complexes to tether the mitochondrial IMM to the OMM [97]. Opa1 also requires Mfn1 to perform the IMM fusion, but the type of Opa1 isoforms that interrelate with Mfn1 and their route of interaction remain unknown [98]. The GTPase action of Opa1 is stimulated by the action of cardiolipin, which allows the complete fusion of the mitochondrial membrane [99].

Post-translational modifications of Opa1 are crucial for the formation of different Opa1 isoforms and its ability to execute fusion. Oma1 and Yme1l are two of the most important protein modifiers of Opa1. Yme1l is a metalloproteinase that acts on the S2 cleavage site of Opa1 [100]. Oma1 processes the cleavage of the S1 site of Opa1 to mediate the formation of S-Opa1 [101]. The balance between the availability of S-Opa1 and L-Opa1 is maintained by the regulatory action of Yme1l on the mature form of Oma1 [102]. This equilibrium is altered under hypoxic conditions, where Oma1 upregulation increases the S-Opa1 isoform and ultimately causes marked fragmentation [103]. In addition, prohibitin (Phb) participates in maintaining the pool of mitochondrial Opa1 isoforms. The defect in Phb decreases the levels of L-Opa1 in IMM, which induces fragmentation [104]. Collectively, the complex pool of proteins that reside in OMM and IMM function together to allow mitochondrial fusion to take place.

### 3.2. Mitochondrial Fission

The segregation of mitochondria from their network is vital for quality control, intracellular dispersion, cell division and death [105,106,107]. Physiological fission starts by sets of constrictions of the mitochondrial inner compartment termed CoMIC that are maintained by the action of S-Opa1. These IMM constrictions are formed by the influx of Ca^2+^ from the ERT, which in turn prompt mitochondrial membrane depolarisation [108]. The oligomerisation and translocation of dynamin-related protein 1 (Drp1) to the ERT-constricted region of OMM is the next step in the process of fission [109]. These regions are created by the inverted formin 2 and spire 1c, which prepare the assembly of F-actin to recruit Drp1 [110,111,112]. The constriction regions are also marked by lysosomal GTP-bound Rab7. Before the OMM translocation of Drp1, fission factor 1 (Fis1) recruits TBC1D15 GTPase to the constriction region and releases lysosomes from mitochondria [113]. Several receptor proteins, including Fis1, mitochondrial fission factor (Mff), as well as Mid49 and Mid51, actively participate in the recruitment of Drp1 [114]. Mid49/51 are suggested to bind to cardiolipin at the OMM/IMM interface and allow the initial recruitment of Drp1 dimers. The tethering of OMM and IMM is achieved by the function of Opa1 [108]. Once bound to the surface of OMM, Drp1 also interacts with Mff to form a higher-order oligomeric complex [115]. The pool of cardiolipin at the OMM then allows the fully assembled Drp1 to instigate OMM fission [116]. Subsequently, S-Opa1 processes IMM fission [100]. Recruitment of Golgi phosphatidylinositol 4-phosphate [PI(4)P] vesicles to the mitochondrial-ERT constriction site has been suggested to be necessary for the progression of fission following Drp 1 action [117]. Additionally, the final steps of mitochondrial division include the action of Dynamin-2, which performs membrane scission and separates the two mitochondria [118]. Despite the comprehensiveness of these findings, the exact nature of S-Opa1, Drp1, PI(4)P and Dynamin-2 cooperation to conclude the final steps of fission is inconclusive. It is noteworthy to mention that Fis1 is also capable of mediating fission independent of Drp1. This action of Fis1 relies on its inhibitory effect on the GTPase activity of mitochondrial fusion proteins [119]. However, further research is required to elucidate whether this type of fission also requires ERT and the aforementioned processes. Besides, it would be intriguing to assess the effects of dual Drp1 and Fis1 knock out (KO) in mitochondrial fission and explore any additional protein machinery that may be involved in this process. One of the potential candidates is Bcl2-L-13, which has been observed to induce fission in a Drp1-independent manner [120]. In this context, additional work is required to understand whether its mechanism of action requires Fis1 or any other alternative fission proteins.

### 3.3. Mitochondrial Fusion and Fission in the Cardiovascular System

Mitochondria occupy around 30% of the cardiomyocyte volume and play a significant role in calcium buffering and β-oxidation required for cardiac contractility, as well as cell death [121]. Investigations on cardiac cells lacking OMM fusion proteins, including Mfn1 and Mfn2, have found surprising effects regarding the linkage of mitochondrial morphology and function. For instance, cardiac-specific ablation of Mfn1 triggers mitochondrial fragmentation. However, its absence does not produce significant changes in cardiomyocyte function [122]. Comparatively, cardiac-specific deletion of Mfn2 causes mitochondrial fragmentation, while enlarging the overall volume of cardiac mitochondria [123,124]. Unlike Mfn1, the absence of Mfn2 in aged animals causes hypertrophy and contractile dysfunction, which are additionally attributed to the pleiotropic role of Mfn2 in mitochondria-sarcoplasmic reticulum tethering [125]. The tethering function of Mfn2 maintains sarcoplasmic reticulum inositol-1,4,5-trisphosphate-related mitochondrial Ca^2+^ uptake to regulate ATP production and cardiac contractility [126]. In comparison with Mfn1 or Mfn2 KO, deletion of both Mfn1 and Mfn2 instigates the depression of cardiac function by reducing stroke volume and output. These effects occur due to altered mitochondrial morphology, diminished state 3 respiration and abrogated mitochondrial Ca^2+^ uptake in cardiac-specific mitofusin 1/2 double KO mice [127]. The *Opa1* gene is abundantly expressed in the heart and its participation in cristae integrity and cell death makes it a necessary gene for embryonic development [128,129]. Opa1^+/−^ mice have marked mitochondrial enlargement, which increases their Ca^2+^ retention capacity. Altered Ca^2+^ handling, in turn, delays the onset of MPTP opening in these hearts. Despite normal respiration and cardiac function in young mice, Opa1^+/−^ cardiac mitochondria have an attenuated energy transfer system to supply the myosin ATPase [129]. This ultimately causes left ventricular dilation and reduced fractional shortening in aged mice [130]. Collectively, the findings in mitofusin and Opa1-deficient mice directly link mitochondrial fusion to cardiac function.

Similar to mitochondrial fusion proteins, cardiac-specific ablation of Drp1 has demonstrated the necessity of mitochondrial fission in the heart [131,132]. The indispensable role of Drp1 in cardiac development can be appreciated by the embryonic lethality of Drp1^−/−^ mice. In the conditional model of Drp1^loxp/loxp^ mice, major clustering of mitochondria, with disparate morphology, occurs in the intracellular space [133]. In addition to impaired fission, this morphological heterogeneity also occurs due to the presence of abrupt mitophagy in Drp1^−/−^ cardiomyocytes [132]. These mitochondria also have a reduced mtDNA copy number, deficient respiration and cristae deformities, which mainly stem from the lack of L-Opa1. These mitochondrial malfunctions, along with disorganised myofibrils, cause a marked dilation of the left ventricle and a subsequent reduction in fractional shortening [133]. Therefore, the balance of mitochondrial morphology that is modulated by endogenous Drp1 is critical for normal cardiac function.

### 3.4. E2 Effects on Mitochondrial Fusion and Fission in the Cardiovascular System

**Figure 2:**
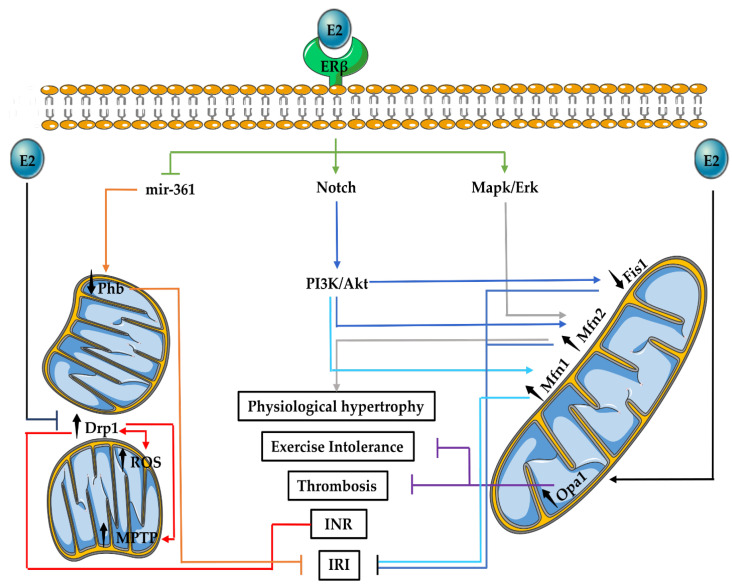
Effects of E2 on mitochondrial dynamics in the cardiovascular system. E2–ERβ interaction modulates the proteins of mitochondrial dynamics in a direct or indirect manner, thereby eliciting effects on cardiovascular (patho)physiology. Abbreviations: Drp1: dynamin-related protein 1, E2: 17β-Oestradiol, ERβ: oestrogen receptor β, Erk: extracellular signal-regulated kinase, Fis1: fission factor 1, INR: insulin resistance, IRI: ischaemia-reperfusion injury, Mapk: mitogen-activated-protein-kinase, Mfn1: mitofusin 1, Mfn2: mitofusin 2, MPTP: mitochondria permeability transition pore, Opa1: optic atrophy 1, PI3K: phosphatidylinositol-3-OH kinase, Phb: prohibitin and ROS: reactive oxygen species.

E2 modulates mitochondrial dynamics exerting regulatory effects in numerous cardiovascular diseases by modifying both fusion and fission proteins. For instance, upon E2 exposure, Mfn2 S-nitrosylation in endothelial cells induces mitochondrial changes that contribute to vascular functions [134]. Hearts of OVX mice have reduced Pgc-1α levels, which in turn may affect the production of both mitochondrial fusion and fission proteins [135,136]. E2 function on cardiac mitochondria has been observed to incorporate ERβ (Figure 2). Female mice develop more pronounced exercise-induced physiological hypertrophy following eight weeks of voluntary cage wheel running in comparison with male mice. These effects were mediated via ERβ and the activation of Mapk/Erk and AKT [137]. Interestingly, exercise led to a shift from large towards smaller mitochondria and a significant reduction in Mfn2 levels only in female mice [137].

In ischaemic injury, ERβ has been observed to rely on Notch 1 signalling upstream of Akt [138]. E2 activation of PI3K/Akt pathway following IRI has been documented to reduce the levels of Fis1 protein and may promote Mfn1-dependent fusion [139,140]. ERβ may also regulate the function of Phb in the setting of IRI. ERβ downregulates the levels of microRNA-361 (mir-361), which is known for reducing the production of Phb, triggering mitochondrial fragmentation following IRI [141,142]. However, while there is some knowledge about the E2-dependent regulation of mitochondrial fission, the effects of E2 on components of mitochondrial fusion during IRI are incompletely understood. In addition, whether components of mitochondrial dynamics play any role in the occurrence of various cell death modalities, including ferroptosis, parthanatos and oxeiptosis, in the setting of IRI, as well as any regulatory effects of E2, remain largely unexplored.

OVX rats receiving a high-fat diet (HFD) have been found to develop insulin resistance, abrupt Ca^2+^ signalling and cardiometabolic dysfunction. These abnormalities occurred in correlation with the impairment of mitochondrial function and dynamics, including mitochondrial fragmentation (due to upregulation of phosphorylated Drp1), increase in reactive oxygen species (ROS) production and higher sensitisation to MPTP stimuli. These effects were partially alleviated by the administration of E2 [143]. In addition, no changes were observed in the levels of mitochondrial fusion proteins, including Opa1, Mfn1 and Mfn2, which suggests preferential effects of E2 on mitochondrial fission in the setting of HFD-induced insulin resistance [143]. Moreover, E2 may participate in anti-thrombotic effects of Opa1. Male and female platelet-specific Opa1 KO have a decrease and increase in platelet-mediated occlusion of the carotid artery, respectively, and this effect was lost in gonadectomized females [144]. Altogether, the effects of E2 in various cardiovascular diseases often involve the inhibition of mitochondrial fission or preservation of mitochondrial shape.

### 3.5. Mitochondrial Fusion and Fission in the Nervous System

Neuronal function is heavily dependent on the function of mitochondria. The generation of ATP for axonal and synaptic function, Ca^2+^-buffering following synaptic neurotransmission and the initiation of cell death are some of the main activities of mitochondria in neurons (reviewed in [145]). Neuronal polarity allows different parts of neurons to have specialised function, structure and mitochondrial distribution. The metabolic state and energy expenditure of different parts of neurons is often provided by mitochondrial transport through these different sections [146]. Mitochondrial transport and localisation in neurons are controlled by the function of mitochondrial fusion and fission proteins. The lack of functional Drp1 has been demonstrated to lower the density of dendritic mitochondria and to disturb mitochondrial movement [147]. In parallel, the overexpression of Opa1 does not alter the density of dendritic mitochondria, while it induces mitochondrial fragmentation. Hence, the alteration of balance of mitochondrial fusion/fission proteins can interfere with dendritic spine formation [147]. CA1 neurons of Opa1^+/−^ mice display reduced spine density. Interestingly, Opa1^+/−^ CA1 neurons of female mice have significantly shorter dendrites and lower ATP production compared with male mice, which together contribute to a distinct form of memory loss [148,149]. The lack of Opa1 has been demonstrated to induce mitochondrial respiratory defects and mitochondrial fragmentation, as well as a reduction in mitochondrial density of neuronal axons in *Drosophila* larvae. These defects could be rescued by the downregulation of Drp1 [150]. Furthermore, Drp1^−/−^ mice die shortly after birth due to neuronal defects. Embryonic stem cells derived from Drp1^−/−^ mice show pronounced mitochondrial filamentation and are more sensitive to staurosporine-mediated apoptosis [151]. Synapses of mutant Drp1 neurons exhibit marked depletion of mitochondria and while being functional at basal states, they fail to maintain neurotransmission following high-frequency stimulation [152]. The sensitivity to apoptosis and neuronal dysfunction in Drp1-depleted neurons can be due to the increased mitochondrial ROS production in hyperfused mitochondria and diminished mitophagy, as well as the loss of mitochondrial respiratory function [153].

Mitofusins also participate in neuronal homeostasis. Unlike Mfn1, ablation of Mfn2 in mice interferes with the growth of Purkinje cells and hampers cerebellum development [154]. In addition, the absence of Mfn2 reduces mitochondrial density in axons and lowers the formation of functional neurons that have fully developed neuronal cell bodies in hippocampal and cortical neurons. In parallel, increased oxidative stress and subsequent apoptosis augment neurodegeneration in the hippocampus and cortex of Mfn2 KO mice [155]. Although deletion of Mfn1 has been observed to be dispensable for the function of Purkinje cells and midbrain dopamine neurons, the increase in its levels in Charcot-Marie-Tooth disease type 2A disease can alleviate motor neuron defects in Mfn2-mutant mice, preventing neurodegeneration and restoring motor function [154,156,157]. Collectively, these studies unravel the importance of balanced mitochondrial dynamics in neuronal function and development.

### 3.6. E2 Effects on Mitochondrial Fusion and Fission in the Nervous System

E2 contributes to neuronal health and function via direct and indirect modulation of mitochondrial dynamics. Ovariectomy and ageing increase mitochondrial fragmentation and the formation of doughnut-shaped mitochondria in monkey prefrontal cortex synapses. This effect was correlated with the loss of working memory and was reversed by E2 treatment [158]. Astrocytes are specialized glial cells present in the central nervous system. These cells regulate numerous cellular functions, including proliferation and cell death, all of which are essential for optimal neuronal function. Cortical astrocytes of male and female mice react differently to E2 in vitro. E2 treatment promotes apoptotic cell death in male astrocytes, while it prevents it in female astrocytes. This effect was attributed to changes in mitochondrial fusion and fission proteins, whereby E2 treatment increased the levels of Mfn2, Drp1 and Fis1 but reduced Mfn1 levels in male astrocytes. The reduction of Mfn1 was correlated with a reduction the levels of anti-apoptotic Bcl2 in these cells [159]. However, the exact mechanism of E2 function on the protein machinery of mitochondrial dynamics in different cells of the nervous system is yet to be fully explored.

**Figure 3:**
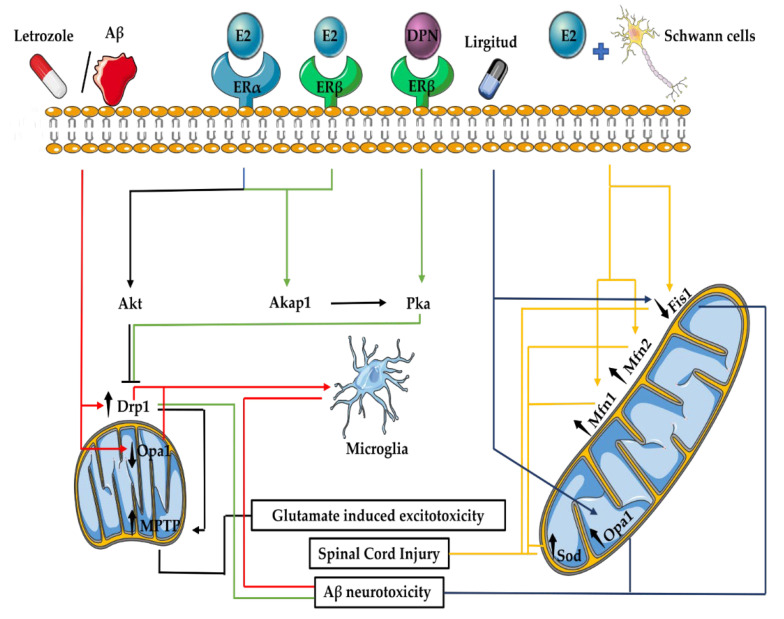
Effects of E2 on mitochondrial dynamics in pathologies of the nervous system. E2-associated signalling via its receptors or drugs that promote E2 production lead to the attenuation of fission and the promotion of fusion. Abbreviations: Aβ: amyloid-beta protein, Akap1: activates kinase anchoring protein 1, Drp1: dynamin-related protein 1, E2: 17β-Oestradiol, ERβ: oestrogen receptor β, Fis1: fission factor 1, Mfn1: mitofusin 1, Mfn2: mitofusin 2, MPTP: mitochondria permeability transition pore, Opa1: optic atrophy 1, Pka: protein kinase A and Sod: sodium oxide dismutase.

In the setting of ischaemia, E2 treatment has been observed to upregulate the levels of total Drp1 in OVX rats with middle cerebral artery occlusion (MCAO). Although E2 treatment alleviated MCAO symptoms in OVX animals, no further explanation was given for the connection between the upregulation of Drp1 and MCAO [160]. Relatively, hypoxic ischaemic male and female brain hemispheres have exhibited increased mitochondrial fragmentation. Mitochondrial fragmentation rescues the female brain following hypoxic ischaemia, as it promotes mitophagy, preventing apoptosis [161]. Conversely, E2 treatment in combination with Schwann cell transplantation promotes recovery following hypoxic ischaemic spinal cord injury via reduction of Fis1 and upregulation of Mfn1 and Mfn2 proteins (see Figure 3). Alteration of mitochondrial fusion proteins also increases the levels of sodium oxide dismutase (Sod), thereby indirectly demonstrating that enhancement of mitochondrial fusion may negatively regulate intracellular ROS during spinal cord injury [162]. Glutamate-induced excitotoxicity, which can occur as a result of hypoxic ischaemia, increases the phosphorylation of Drp1 at its serine 616 and promotes mitochondrial fragmentation and MPTP opening in HT22 hippocampal cells. E2 treatment blocks glutamate-induced excitotoxicity. This effect has been attributed to the utilisation of the Akt pathway promoting the survival of HT22 hippocampal cells [163,164].

E2 and its receptors may also provide therapeutic benefits in other diseases. Leber’s hereditary optic neuropathy leads to significant vision loss. Treatment of Leber’s hereditary optic neuropathy osteosarcoma-derived cybrid cells with an ER antagonist (ICI 182780) leads to excessive ROS production, mitochondrial membrane dissipation and cell death. These effects were correlated with marked mitochondrial fragmentation and could be rescued by E2 treatment [165]. ERβ associates and activates the kinase-anchoring protein 1 (Akap1), which in turn binds to Pka and anchors this protein to the OMM. Pka then phosphorylates Drp1 at serine 637 and inhibits its fission activity. In a model of Alzheimer’s disease (AD) involving primary hippocampal neurons, Aβ treatment increases mitochondrial fission, disturbs mitochondrial transport and mitochondrial oxygen consumption rate. An ERβ agonist (DPN) reversed the effects of Aβ and promoted the phosphorylation of Drp1 by Pka, which in turn induced mitochondrial elongation and prevented Aβ neurotoxicity [166]. Chronic E2 deficiency in letrozole-treated or OVX mice carrying the β-amyloid precursor protein transgene causes mitochondrial respiratory defects and mitochondrial fragmentation in residential central nervous system macrophage populations (microglia). This is due to the increase in Drp1 and decrease in Opa1 levels, which ultimately promote microglia activation [167,168]. Exogenous E2 administration can reverse these effects and prevent microglial activation [168]. Targeted therapeutics against AD also benefit from the link between E2 and mitochondrial dynamics. For instance, in a 3xTg-AD mouse model, which develops early AD-like symptoms, Liraglutide restored glucose metabolism and brain E2 levels. This, in turn, reversed the decrease and increase in the levels of Opa1 and Fis1, respectively, and normalised brain oxidative markers [169]. Therefore, E2-mediated preservation of mitochondrial shape, as well as the reduction of mitochondrial fission, may offer therapeutic benefit in neurological and neurodegenerative diseases.

## 4. Potential Interplay of E2 and Mitochondrial Dynamics in the Heart–Brain Axis

The bidirectional synergistic relationship between the cardiovascular and nervous systems and their respective homeostasis have been recently put forward in the concept of HBA [170,171]. This axis incorporates the effects of cardiovascular diseases on the nervous system and effects of neurological disorders on the cardiovascular system. The effects of cardiovascular disease on the nervous system have been widely studied. However, the effects of neurological disorders on the cardiovascular system are poorly understood. Various pathologies of the nervous system can lead to a wide range of alterations in function and structure of the cardiovascular system, ranging from transient and benign electrographic changes to myocardial injury, cardiomyopathy and even cardiac death [170].

For instance, patients develop electrocardiographic abnormalities and left ventricular dysfunction following brain injury [172]. Production of higher levels of Aβ increases the susceptibility to AD in patients suffering from hypertension [173]. Individuals who experience myocardial infarction have a higher risk of developing vascular dementia [174]. Similarly, acute stroke has been suggested to increase the risk of cardiovascular diseases [175]. While neurotransmitters, such as catecholamines, participate in the HBA (reviewed in [176]), the exact signalling pathways of HBA under normal and diseased conditions remain unknown.

**Figure 4:**
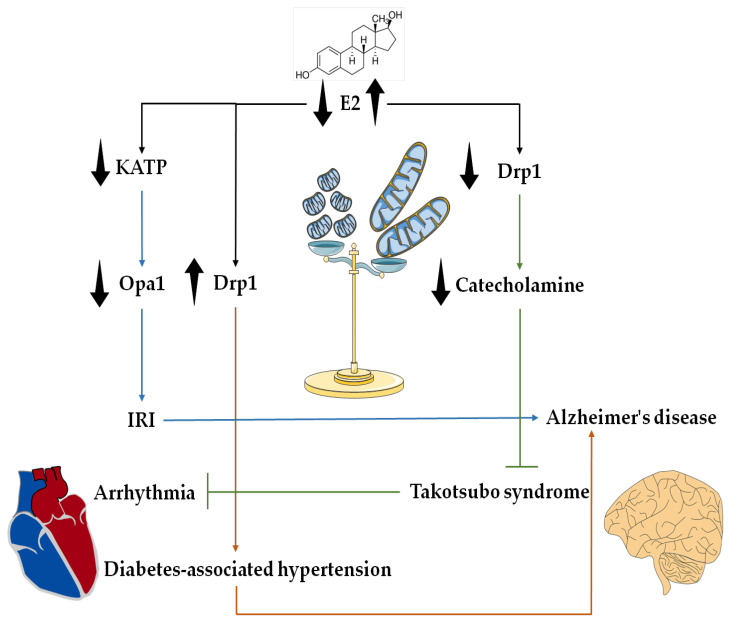
The significance of E2 regulation of mitochondrial dynamics in the heart–brain axis (HBA). Current literature points towards the attenuating effects of E2 on mitochondrial fission. In particular, Drp1 inhibition is involved in the effects of E2 in HBA, as the absence of E2 leads to increased fission or decreased fusion. These alterations then promote cardiovascular diseases that are linked to Alzheimer’s disease. On the other hand, the presence of E2 may potentially inhibit the fission activity of Drp1 to prevent cardiovascular diseases that stem from neurological pathologies.

The lack of E2 and its regulatory effects on components of mitochondrial dynamics may be associated with worse outcomes in the HBA (Figure 4). OVX mice lacking the integral component of mitochondrial potassium ATP channel known as SRU2 subunit have decreased levels of Opa1, ERα and ERβ and display significant left ventricular dysfunction, as well as larger infarcts compared with their WT littermates. E2 treatment was insufficient to reverse these effects in SRU2 KO hearts, thereby suggesting that the E2 preservation of mitochondrial dynamics in the setting of IRI incorporates the N-glycosylation of the mitochondrial potassium ATP channels. Interestingly, KO mice demonstrated enrichment in pathways related to neurodegeneration post IRI, thereby linking the effects of E2 on mitochondrial dynamics with the potential induction of neurological dysfunction and neurodegeneration [177].

One of the major comorbidities that affect cardiac function is diabetes. E2 has been found to be involved in the development of insulin resistance and obesity. OVX Wistar rats that were given a HFD had faster weight gain in comparison with intact rats receiving a HFD or normal diet, or OVX rats receiving a normal diet. In addition, compared with control rats, all other groups developed insulin resistance. HFD OVX rats developed the worst cardiac dysfunction with both systolic and diastolic hypertension, as well as reduced fractional shortening compared with the other groups. Cardiac dysfunction occurred as a result of mitochondrial impairment, whereby cardiac mitochondria of HFD OVX mice had the highest levels of ROS production, mitochondrial swelling and sensitivity to apoptotic cell death compared with the other groups. The mitochondrial abnormalities in this group occurred in parallel to increased mitochondrial fission due to upregulated phosphorylation of Drp1 at its serine 616 residue, thereby linking the E2 deprivation-induced mitochondrial fission to the development of diabetes-related cardiac symptoms [178]. As there is a causal link between the development of AD from hypertension, it is plausible to consider the potential involvement of E2 and Drp1 interaction in the pathogenesis of AD that stems from the diabetes-related cardiac dysfunction [179,180].

Comparatively, the interplay of E2 with components of mitochondrial dynamics may also participate in neurological and neurodegenenerative diseases that cause cardiac disorders. For instance, in Takotsubo syndrome, a potent release of catecholamine from adrenal glands and postganglionic neurons of the sympathetic nervous system into the bloodstream mediates the induction of cardiomyopathy. While the underlying pathogenesis of this syndrome remains unknown, its occurrence is higher in postmenopausal women [181,182]. A study on human induced pluripotent stem cell-derived cardiomyocytes demonstrated that catecholamine treatment induced arrhythmia in these cells via β-adrenergic receptors and upregulation of cellular ROS levels. E2 treatment reduced ROS levels and β-adrenergic receptor activity, thereby inhibiting the toxic effects of catecholamine release [183]. Catecholamine has been demonstrated to cause cardiomyocyte death via MPTP opening. The mechanism of catecholamine-mediated cell death incorporates the elevation of ROS and phosphorylation of Drp1 protein at its serine 616 site via CamKII, thereby suggesting a potential role of mitochondria fission in the induction of Takotsubo syndrome [184]. Considering the inhibitory effect of E2 on Drp1 phosphorylation, E2 may reverse catecholamine-induced cardiotoxicity via downregulation of the Drp1-induced fission. These results hint towards the potential significance of E2 modulation of Drp1 and mitochondrial fission in HBA-related pathologies. However, further research is necessary to delineate the exact participation of mitochondrial fusion and fission components in this concept. Overall, the impact of E2, as well as biological sex, on HBA dysfunction opens unexpected new avenues for further research and the management of both male and female patients.

## 5. Conclusions

E2 exerts direct and indirect effects to regulate mitochondrial dynamics in both the cardiovascular and nervous systems. The effects of E2 mainly encompass the attenuation of mitochondrial fission by targeting Drp1, as well as the preservation of mitochondrial fusion proteins. This cross-talk between E2 and components of mitochondrial dynamics is necessary for cell survival. Finally, the interplay of E2 with mitochondrial dynamics may be a potential therapeutic target in cardiovascular diseases and nervous system-related disorders, as well as in the context of HBA. However, further research is required to shed light into the exact nature of this and to better understand the underlying mechanisms.

## Data Availability

Not applicable.

## References

[1] Gaignebet L., Kararigas G. (2017). En route to precision medicine through the integration of biological sex into pharmacogenomics. Clin. Sci..

[2] Kararigas G., Seeland U., de Arellano M.L.B., Dworatzek E., Regitz-Zagrosek V. (2016). Why the study of the effects of biological sex is important. Commentary. Ann. Ist. Super. Sanita.

[3] Cui C., Huang C., Liu K., Xu G., Yang J., Zhou Y., Feng Y., Kararigas G., Geng B., Cui Q. (2018). Large-scale in silico identification of drugs exerting sex-specific effects in the heart. J. Transl. Med..

[4] Ruiz-Meana M., Boengler K., Garcia-Dorado D., Hausenloy D.J., Kaambre T., Kararigas G., Perrino C., Schulz R., Ytrehus K. (2020). Ageing, sex, and cardioprotection. Br. J. Pharmacol..

[5] Altinbas L., Bormann N., Lehmann D., Jeuthe S., Wulsten D., Kornak U., Robinson P.N., Wildemann B., Kararigas G. (2019). Assessment of Bones Deficient in Fibrillin-1 Microfibrils Reveals Pronounced Sex Differences. Int. J. Mol. Sci..

[6] Li S., Kararigas G. (2022). Role of Biological Sex in the Cardiovascular-Gut Microbiome Axis. Front. Cardiovasc. Med..

[7] Regitz-Zagrosek V., Kararigas G. (2017). Mechanistic Pathways of Sex Differences in Cardiovascular Disease. Physiol. Rev..

[8] Ober C., Loisel D.A., Gilad Y. (2008). Sex-specific genetic architecture of human disease. Nat. Rev. Genet..

[9] McCarthy M.M., Nugent B.M., Lenz K.M. (2017). Neuroimmunology and neuroepigenetics in the establishment of sex differences in the brain. Nat. Rev. Neurosci..

[10] Kokras N., Hodes G.E., Bangasser D.A., Dalla C. (2019). Sex differences in the hypothalamic-pituitary-adrenal axis: An obstacle to antidepressant drug development?. Br. J. Pharmacol..

[11] Kararigas G., Dworatzek E., Petrov G., Summer H., Schulze T.M., Baczko I., Knosalla C., Golz S., Hetzer R., Regitz-Zagrosek V. (2014). Sex-dependent regulation of fibrosis and inflammation in human left ventricular remodelling under pressure overload. Eur. J. Heart Fail..

[12] Gaignebet L., Kandula M.M., Lehmann D., Knosalla C., Kreil D.P., Kararigas G. (2020). Sex-Specific Human Cardiomyocyte Gene Regulation in Left Ventricular Pressure Overload. Mayo Clin. Proc..

[13] Dworatzek E., Baczko I., Kararigas G. (2016). Effects of aging on cardiac extracellular matrix in men and women. Proteom.-Clin. Appl..

[14] Petrov G., Dworatzek E., Schulze T.M., Dandel M., Kararigas G., Mahmoodzadeh S., Knosalla C., Hetzer R., Regitz-Zagrosek V. (2014). Maladaptive remodeling is associated with impaired survival in women but not in men after aortic valve replacement. JACC Cardiovasc. Imaging.

[15] Pei J., Harakalova M., Treibel T.A., Lumbers R.T., Boukens B.J., Efimov I.R., van Dinter J.T., Gonzalez A., Lopez B., El Azzouzi H. (2020). H3K27ac acetylome signatures reveal the epigenomic reorganization in remodeled non-failing human hearts. Clin. Epigenet..

[16] Iorga A., Cunningham C.M., Moazeni S., Ruffenach G., Umar S., Eghbali M. (2017). The protective role of estrogen and estrogen receptors in cardiovascular disease and the controversial use of estrogen therapy. Biol. Sex Differ..

[17] Murphy E. (2011). Estrogen signaling and cardiovascular disease. Circ. Res..

[18] Murphy E., Steenbergen C. (2014). Estrogen regulation of protein expression and signaling pathways in the heart. Biol. Sex Differ..

[19] Menazza S., Murphy E. (2016). The Expanding Complexity of Estrogen Receptor Signaling in the Cardiovascular System. Circ. Res..

[20] Puglisi R., Mattia G., Care A., Marano G., Malorni W., Matarrese P. (2019). Non-genomic Effects of Estrogen on Cell Homeostasis and Remodeling With Special Focus on Cardiac Ischemia/Reperfusion Injury. Front. Endocrinol..

[21] Lowe D.A., Kararigas G. (2020). Editorial: New Insights into Estrogen/Estrogen Receptor Effects in the Cardiac and Skeletal Muscle. Front. Endocrinol..

[22] Kararigas G. (2021). Oestrogenic contribution to sex-biased left ventricular remodelling: The male implication. Int. J. Cardiol..

[23] Schubert C., Raparelli V., Westphal C., Dworatzek E., Petrov G., Kararigas G., Regitz-Zagrosek V. (2016). Reduction of apoptosis and preservation of mitochondrial integrity under ischemia/reperfusion injury is mediated by estrogen receptor beta. Biol. Sex Differ..

[24] Mahmoodzadeh S., Dworatzek E. (2019). The Role of 17beta-Estradiol and Estrogen Receptors in Regulation of Ca(2+) Channels and Mitochondrial Function in Cardiomyocytes. Front. Endocrinol..

[25] Sickinghe A.A., Korporaal S.J.A., den Ruijter H.M., Kessler E.L. (2019). Estrogen Contributions to Microvascular Dysfunction Evolving to Heart Failure With Preserved Ejection Fraction. Front. Endocrinol..

[26] Ventura-Clapier R., Piquereau J., Veksler V., Garnier A. (2019). Estrogens, Estrogen Receptors Effects on Cardiac and Skeletal Muscle Mitochondria. Front. Endocrinol..

[27] Zhang B., Miller V.M., Miller J.D. (2019). Influences of Sex and Estrogen in Arterial and Valvular Calcification. Front. Endocrinol..

[28] Kararigas G., Fliegner D., Forler S., Klein O., Schubert C., Gustafsson J.A., Klose J., Regitz-Zagrosek V. (2014). Comparative Proteomic Analysis Reveals Sex and Estrogen Receptor beta Effects in the Pressure Overloaded Heart. J. Proteome Res..

[29] Kararigas G., Fliegner D., Gustafsson J.A., Regitz-Zagrosek V. (2011). Role of the estrogen/estrogen-receptor-beta axis in the genomic response to pressure overload-induced hypertrophy. Physiol. Genom..

[30] Kararigas G., Nguyen B.T., Jarry H. (2014). Estrogen modulates cardiac growth through an estrogen receptor alpha-dependent mechanism in healthy ovariectomized mice. Mol. Cell. Endocrinol..

[31] Kararigas G., Nguyen B.T., Zelarayan L.C., Hassenpflug M., Toischer K., Sanchez-Ruderisch H., Hasenfuss G., Bergmann M.W., Jarry H., Regitz-Zagrosek V. (2014). Genetic background defines the regulation of postnatal cardiac growth by 17beta-estradiol through a beta-catenin mechanism. Endocrinology.

[32] Sanchez-Ruderisch H., Queiros A.M., Fliegner D., Eschen C., Kararigas G., Regitz-Zagrosek V. (2019). Sex-specific regulation of cardiac microRNAs targeting mitochondrial proteins in pressure overload. Biol. Sex Differ..

[33] Duft K., Schanz M., Pham H., Abdelwahab A., Schriever C., Kararigas G., Dworatzek E., Davidson M.M., Regitz-Zagrosek V., Morano I. (2017). 17beta-Estradiol-induced interaction of estrogen receptor alpha and human atrial essential myosin light chain modulates cardiac contractile function. Basic Res. Cardiol..

[34] Lai S., Collins B.C., Colson B.A., Kararigas G., Lowe D.A. (2016). Estradiol modulates myosin regulatory light chain phosphorylation and contractility in skeletal muscle of female mice. Am. J. Physiol. Endocrinol. Metab..

[35] Mahmoodzadeh S., Pham T.H., Kuehne A., Fielitz B., Dworatzek E., Kararigas G., Petrov G., Davidson M.M., Regitz-Zagrosek V. (2012). 17beta-Estradiol-induced interaction of ERalpha with NPPA regulates gene expression in cardiomyocytes. Cardiovasc. Res..

[36] Nguyen B.T., Kararigas G., Jarry H. (2012). Dose-dependent effects of a genistein-enriched diet in the heart of ovariectomized mice. Genes Nutr..

[37] Nguyen B.T., Kararigas G., Wuttke W., Jarry H. (2012). Long-term treatment of ovariectomized mice with estradiol or phytoestrogens as a new model to study the role of estrogenic substances in the heart. Planta Med..

[38] Szego E.M., Barabas K., Balog J., Szilagyi N., Korach K.S., Juhasz G., Abraham I.M. (2006). Estrogen induces estrogen receptor alpha-dependent cAMP response element-binding protein phosphorylation via mitogen activated protein kinase pathway in basal forebrain cholinergic neurons in vivo. J. Neurosci..

[39] Sabbatini A.R., Kararigas G. (2020). Menopause-Related Estrogen Decrease and the Pathogenesis of HFpEF: JACC Review Topic of the Week. J. Am. Coll. Cardiol..

[40] Sabbatini A.R., Kararigas G. (2020). Estrogen-related mechanisms in sex differences of hypertension and target organ damage. Biol. Sex Differ..

[41] Kararigas G., Becher E., Mahmoodzadeh S., Knosalla C., Hetzer R., Regitz-Zagrosek V. (2010). Sex-specific modification of progesterone receptor expression by 17beta-oestradiol in human cardiac tissues. Biol. Sex Differ..

[42] Kararigas G., Bito V., Tinel H., Becher E., Baczko I., Knosalla C., Albrecht-Kupper B., Sipido K.R., Regitz-Zagrosek V. (2012). Transcriptome characterization of estrogen-treated human myocardium identifies Myosin regulatory light chain interacting protein as a sex-specific element influencing contractile function. J. Am. Coll. Cardiol..

[43] Hein S., Hassel D., Kararigas G. (2019). The Zebrafish (Danio rerio) Is a Relevant Model for Studying Sex-Specific Effects of 17beta-Estradiol in the Adult Heart. Int. J. Mol. Sci..

[44] Fliegner D., Schubert C., Penkalla A., Witt H., Kararigas G., Dworatzek E., Staub E., Martus P., Ruiz Noppinger P., Kintscher U. (2010). Female sex and estrogen receptor-beta attenuate cardiac remodeling and apoptosis in pressure overload. Am. J. Physiol. Regul. Integr. Comp. Physiol..

[45] Queiros A.M., Eschen C., Fliegner D., Kararigas G., Dworatzek E., Westphal C., Sanchez Ruderisch H., Regitz-Zagrosek V. (2013). Sex- and estrogen-dependent regulation of a miRNA network in the healthy and hypertrophied heart. Int. J. Cardiol..

[46] Murray H.E., Pillai A.V., McArthur S.R., Razvi N., Datla K.P., Dexter D.T., Gillies G.E. (2003). Dose- and sex-dependent effects of the neurotoxin 6-hydroxydopamine on the nigrostriatal dopaminergic pathway of adult rats: Differential actions of estrogen in males and females. Neuroscience.

[47] Klinge C.M. (2020). Estrogenic control of mitochondrial function. Redox Biol..

[48] Luo T., Liu H., Kim J.K. (2016). Estrogen Protects the Female Heart from Ischemia/Reperfusion Injury through Manganese Superoxide Dismutase Phosphorylation by Mitochondrial p38beta at Threonine 79 and Serine 106. PLoS ONE.

[49] Tsialtas I., Georgantopoulos A., Karipidou M.E., Kalousi F.D., Karra A.G., Leonidas D.D., Psarra A.G. (2021). Anti-Apoptotic and Antioxidant Activities of the Mitochondrial Estrogen Receptor Beta in N2A Neuroblastoma Cells. Int. J. Mol. Sci..

[50] Zhai P., Eurell T.E., Cotthaus R., Jeffery E.H., Bahr J.M., Gross D.R. (2000). Effect of estrogen on global myocardial ischemia-reperfusion injury in female rats. Am. J. Physiol. Heart Circ. Physiol..

[51] Sasaki Y., Ikeda Y., Uchikado Y., Akasaki Y., Sadoshima J., Ohishi M. (2021). Estrogen Plays a Crucial Role in Rab9-Dependent Mitochondrial Autophagy, Delaying Arterial Senescence. J. Am. Heart Assoc..

[52] Zhao W., Hou Y., Song X., Wang L., Zhang F., Zhang H., Yu H., Zhou Y. (2021). Estrogen Deficiency Induces Mitochondrial Damage Prior to Emergence of Cognitive Deficits in a Postmenopausal Mouse Model. Front. Aging Neurosci..

[53] Octavia Y., Kararigas G., de Boer M., Chrifi I., Kietadisorn R., Swinnen M., Duimel H., Verheyen F.K., Brandt M.M., Fliegner D. (2017). Folic acid reduces doxorubicin-induced cardiomyopathy by modulating endothelial nitric oxide synthase. J. Cell. Mol. Med..

[54] Zhao R.Z., Jiang S., Zhang L., Yu Z.B. (2019). Mitochondrial electron transport chain, ROS generation and uncoupling (Review). Int. J. Mol. Med..

[55] Hazell G.G., Yao S.T., Roper J.A., Prossnitz E.R., O’Carroll A.M., Lolait S.J. (2009). Localisation of GPR30, a novel G protein-coupled oestrogen receptor, suggests multiple functions in rodent brain and peripheral tissues. J. Endocrinol..

[56] Stirone C., Duckles S.P., Krause D.N., Procaccio V. (2005). Estrogen increases mitochondrial efficiency and reduces oxidative stress in cerebral blood vessels. Mol. Pharmacol..

[57] Yang S.H., Liu R., Perez E.J., Wen Y., Stevens S.M., Valencia T., Brun-Zinkernagel A.M., Prokai L., Will Y., Dykens J. (2004). Mitochondrial localization of estrogen receptor beta. Proc. Natl. Acad. Sci. USA.

[58] Pugach E.K., Blenck C.L., Dragavon J.M., Langer S.J., Leinwand L.A. (2016). Estrogen receptor profiling and activity in cardiac myocytes. Mol. Cell. Endocrinol..

[59] He F., Ru X., Wen T. (2020). NRF2, a Transcription Factor for Stress Response and Beyond. Int. J. Mol. Sci..

[60] Maher A.C., Akhtar M., Tarnopolsky M.A. (2010). Men supplemented with 17beta-estradiol have increased beta-oxidation capacity in skeletal muscle. Physiol. Genom..

[61] Wu Z., Puigserver P., Andersson U., Zhang C., Adelmant G., Mootha V., Troy A., Cinti S., Lowell B., Scarpulla R.C. (1999). Mechanisms controlling mitochondrial biogenesis and respiration through the thermogenic coactivator PGC-1. Cell.

[62] Rius-Perez S., Torres-Cuevas I., Millan I., Ortega A.L., Perez S. (2020). PGC-1alpha, Inflammation, and Oxidative Stress: An Integrative View in Metabolism. Oxid. Med. Cell Longev..

[63] Chen G., Kroemer G., Kepp O. (2020). Mitophagy: An Emerging Role in Aging and Age-Associated Diseases. Front. Cell Dev. Biol..

[64] Marinkovic M., Sprung M., Novak I. (2021). Dimerization of mitophagy receptor BNIP3L/NIX is essential for recruitment of autophagic machinery. Autophagy.

[65] Liu L., Feng D., Chen G., Chen M., Zheng Q., Song P., Ma Q., Zhu C., Wang R., Qi W. (2012). Mitochondrial outer-membrane protein FUNDC1 mediates hypoxia-induced mitophagy in mammalian cells. Nat. Cell Biol..

[66] Chen G., Han Z., Feng D., Chen Y., Chen L., Wu H., Huang L., Zhou C., Cai X., Fu C. (2014). A regulatory signaling loop comprising the PGAM5 phosphatase and CK2 controls receptor-mediated mitophagy. Mol. Cell.

[67] Shiba-Fukushima K., Imai Y., Yoshida S., Ishihama Y., Kanao T., Sato S., Hattori N. (2012). PINK1-mediated phosphorylation of the Parkin ubiquitin-like domain primes mitochondrial translocation of Parkin and regulates mitophagy. Sci. Rep..

[68] Burton T.R., Gibson S.B. (2009). The role of Bcl-2 family member BNIP3 in cell death and disease: NIPping at the heels of cell death. Cell Death Differ..

[69] Chen B.C., Weng Y.J., Shibu M.A., Han C.K., Chen Y.S., Shen C.Y., Lin Y.M., Viswanadha V.P., Liang H.Y., Huang C.Y. (2018). Estrogen and/or Estrogen Receptor alpha Inhibits BNIP3-Induced Apoptosis and Autophagy in H9c2 Cardiomyoblast Cells. Int. J. Mol. Sci..

[70] Yao M., Nguyen T.V., Pike C.J. (2007). Estrogen regulates Bcl-w and Bim expression: Role in protection against beta-amyloid peptide-induced neuronal death. J. Neurosci..

[71] Hsieh D.J., Kuo W.W., Lai Y.P., Shibu M.A., Shen C.Y., Pai P., Yeh Y.L., Lin J.Y., Viswanadha V.P., Huang C.Y. (2015). 17beta-Estradiol and/or Estrogen Receptor beta Attenuate the Autophagic and Apoptotic Effects Induced by Prolonged Hypoxia Through HIF-1alpha-Mediated BNIP3 and IGFBP-3 Signaling Blockage. Cell Physiol. Biochem..

[72] Feng Y., Madungwe N.B., da Cruz Junho C.V., Bopassa J.C. (2017). Activation of G protein-coupled oestrogen receptor 1 at the onset of reperfusion protects the myocardium against ischemia/reperfusion injury by reducing mitochondrial dysfunction and mitophagy. Br. J. Pharmacol..

[73] Gureev A.P., Shaforostova E.A., Popov V.N. (2019). Regulation of Mitochondrial Biogenesis as a Way for Active Longevity: Interaction Between the Nrf2 and PGC-1alpha Signaling Pathways. Front. Genet..

[74] Capllonch-Amer G., Sbert-Roig M., Galmes-Pascual B.M., Proenza A.M., Llado I., Gianotti M., Garcia-Palmer F.J. (2014). Estradiol stimulates mitochondrial biogenesis and adiponectin expression in skeletal muscle. J. Endocrinol..

[75] Kemper M.F., Stirone C., Krause D.N., Duckles S.P., Procaccio V. (2014). Genomic and non-genomic regulation of PGC1 isoforms by estrogen to increase cerebral vascular mitochondrial biogenesis and reactive oxygen species protection. Eur. J. Pharmacol..

[76] Galmes-Pascual B.M., Nadal-Casellas A., Bauza-Thorbrugge M., Sbert-Roig M., Garcia-Palmer F.J., Proenza A.M., Gianotti M., Llado I. (2017). 17beta-estradiol improves hepatic mitochondrial biogenesis and function through PGC1B. J. Endocrinol..

[77] Sbert-Roig M., Bauza-Thorbrugge M., Galmes-Pascual B.M., Capllonch-Amer G., Garcia-Palmer F.J., Llado I., Proenza A.M., Gianotti M. (2016). GPER mediates the effects of 17beta-estradiol in cardiac mitochondrial biogenesis and function. Mol. Cell. Endocrinol..

[78] Tait S.W., Green D.R. (2013). Mitochondrial regulation of cell death. Cold Spring Harb. Perspect. Biol..

[79] Wang H., Yu S.W., Koh D.W., Lew J., Coombs C., Bowers W., Federoff H.J., Poirier G.G., Dawson T.M., Dawson V.L. (2004). Apoptosis-inducing factor substitutes for caspase executioners in NMDA-triggered excitotoxic neuronal death. J. Neurosci..

[80] Xu T., Ding W., Tariq M.A., Wang Y., Wan Q., Li M., Wang J. (2018). Molecular mechanism and therapy application of necrosis during myocardial injury. J. Cell. Mol. Med..

[81] Milerova M., Drahota Z., Chytilova A., Tauchmannova K., Houstek J., Ostadal B. (2016). Sex difference in the sensitivity of cardiac mitochondrial permeability transition pore to calcium load. Mol. Cell Biochem..

[82] Mendelowitsch A., Ritz M.F., Ros J., Langemann H., Gratzl O. (2001). 17beta-Estradiol reduces cortical lesion size in the glutamate excitotoxicity model by enhancing extracellular lactate: A new neuroprotective pathway. Brain Res..

[83] Jover-Mengual T., Miyawaki T., Latuszek A., Alborch E., Zukin R.S., Etgen A.M. (2010). Acute estradiol protects CA1 neurons from ischemia-induced apoptotic cell death via the PI3K/Akt pathway. Brain Res..

[84] Patten R.D., Pourati I., Aronovitz M.J., Baur J., Celestin F., Chen X., Michael A., Haq S., Nuedling S., Grohe C. (2004). 17beta-estradiol reduces cardiomyocyte apoptosis in vivo and in vitro via activation of phospho-inositide-3 kinase/Akt signaling. Circ. Res..

[85] Pelzer T., Neumann M., de Jager T., Jazbutyte V., Neyses L. (2001). Estrogen effects in the myocardium: Inhibition of NF-kappaB DNA binding by estrogen receptor-alpha and -beta. Biochem. Biophys. Res. Commun..

[86] Waters E.M., Simerly R.B. (2009). Estrogen induces caspase-dependent cell death during hypothalamic development. J. Neurosci..

[87] Batnasan E., Wang R., Wen J., Ke Y., Li X., Bohio A.A., Zeng X., Huo H., Han L., Boldogh I. (2015). 17-beta estradiol inhibits oxidative stress-induced accumulation of AIF into nucleolus and PARP1-dependent cell death via estrogen receptor alpha. Toxicol. Lett..

[88] Kalkhoran S.B., Hernandez-Resendiz S., Ong S.G., Ramachandra C.J.A., Hausenloy D.J. (2020). Mitochondrial shaping proteins as novel treatment targets for cardiomyopathies. Cond. Med..

[89] Chen H., Vermulst M., Wang Y.E., Chomyn A., Prolla T.A., McCaffery J.M., Chan D.C. (2010). Mitochondrial fusion is required for mtDNA stability in skeletal muscle and tolerance of mtDNA mutations. Cell.

[90] Silva Ramos E., Motori E., Bruser C., Kuhl I., Yeroslaviz A., Ruzzenente B., Kauppila J.H.K., Busch J.D., Hultenby K., Habermann B.H. (2019). Mitochondrial fusion is required for regulation of mitochondrial DNA replication. PLoS Genet..

[91] Tondera D., Grandemange S., Jourdain A., Karbowski M., Mattenberger Y., Herzig S., Da Cruz S., Clerc P., Raschke I., Merkwirth C. (2009). SLP-2 is required for stress-induced mitochondrial hyperfusion. EMBO J..

[92] Chen H., Detmer S.A., Ewald A.J., Griffin E.E., Fraser S.E., Chan D.C. (2003). Mitofusins Mfn1 and Mfn2 coordinately regulate mitochondrial fusion and are essential for embryonic development. J. Cell Biol..

[93] Daste F., Sauvanet C., Bavdek A., Baye J., Pierre F., Le Borgne R., David C., Rojo M., Fuchs P., Tareste D. (2018). The heptad repeat domain 1 of Mitofusin has membrane destabilization function in mitochondrial fusion. EMBO Rep..

[94] Zhang Y., Liu X., Bai J., Tian X., Zhao X., Liu W., Duan X., Shang W., Fan H.Y., Tong C. (2016). Mitoguardin Regulates Mitochondrial Fusion through MitoPLD and Is Required for Neuronal Homeostasis. Mol. Cell.

[95] Choi S.Y., Huang P., Jenkins G.M., Chan D.C., Schiller J., Frohman M.A. (2006). A common lipid links Mfn-mediated mitochondrial fusion and SNARE-regulated exocytosis. Nat. Cell Biol..

[96] Head B., Griparic L., Amiri M., Gandre-Babbe S., van der Bliek A.M. (2009). Inducible proteolytic inactivation of OPA1 mediated by the OMA1 protease in mammalian cells. J. Cell Biol..

[97] Ge Y., Shi X., Boopathy S., McDonald J., Smith A.W., Chao L.H. (2020). Two forms of Opa1 cooperate to complete fusion of the mitochondrial inner-membrane. eLife.

[98] Cipolat S., Martins de Brito O., Dal Zilio B., Scorrano L. (2004). OPA1 requires mitofusin 1 to promote mitochondrial fusion. Proc. Natl. Acad. Sci. USA.

[99] Ban T., Ishihara T., Kohno H., Saita S., Ichimura A., Maenaka K., Oka T., Mihara K., Ishihara N. (2017). Molecular basis of selective mitochondrial fusion by heterotypic action between OPA1 and cardiolipin. Nat. Cell Biol..

[100] Anand R., Wai T., Baker M.J., Kladt N., Schauss A.C., Rugarli E., Langer T. (2014). The i-AAA protease YME1L and OMA1 cleave OPA1 to balance mitochondrial fusion and fission. J. Cell Biol..

[101] Ehses S., Raschke I., Mancuso G., Bernacchia A., Geimer S., Tondera D., Martinou J.C., Westermann B., Rugarli E.I., Langer T. (2009). Regulation of OPA1 processing and mitochondrial fusion by m-AAA protease isoenzymes and OMA1. J. Cell Biol..

[102] Consolato F., Maltecca F., Tulli S., Sambri I., Casari G. (2018). m-AAA and i-AAA complexes coordinate to regulate OMA1, the stress-activated supervisor of mitochondrial dynamics. J. Cell Sci..

[103] Nan J., Nan C., Ye J., Qian L., Geng Y., Xing D., Rahman M.S.U., Huang M. (2019). EGCG protects cardiomyocytes against hypoxia-reperfusion injury through inhibition of OMA1 activation. J. Cell Sci..

[104] Merkwirth C., Dargazanli S., Tatsuta T., Geimer S., Lower B., Wunderlich F.T., von Kleist-Retzow J.C., Waisman A., Westermann B., Langer T. (2008). Prohibitins control cell proliferation and apoptosis by regulating OPA1-dependent cristae morphogenesis in mitochondria. Genes Dev..

[105] Zhang H., Sheu S.-S., Wang W. (2014). Fission Promotes Respiration and ROS Production in Individual Mitochondria. Biophys. J..

[106] Smirnova E., Griparic L., Shurland D.L., van der Bliek A.M. (2001). Dynamin-related protein Drp1 is required for mitochondrial division in mammalian cells. Mol. Biol. Cell.

[107] Smirnova E., Shurland D.L., Ryazantsev S.N., van der Bliek A.M. (1998). A human dynamin-related protein controls the distribution of mitochondria. J. Cell Biol..

[108] Cho B., Cho H.M., Jo Y., Kim H.D., Song M., Moon C., Kim H., Kim K., Sesaki H., Rhyu I.J. (2017). Constriction of the mitochondrial inner compartment is a priming event for mitochondrial division. Nat. Commun..

[109] Li S., Xu S., Roelofs B.A., Boyman L., Lederer W.J., Sesaki H., Karbowski M. (2015). Transient assembly of F-actin on the outer mitochondrial membrane contributes to mitochondrial fission. J. Cell Biol..

[110] Manor U., Bartholomew S., Golani G., Christenson E., Kozlov M., Higgs H., Spudich J., Lippincott-Schwartz J. (2015). A mitochondria-anchored isoform of the actin-nucleating spire protein regulates mitochondrial division. eLife.

[111] Chakrabarti R., Ji W.K., Stan R.V., de Juan Sanz J., Ryan T.A., Higgs H.N. (2018). INF2-mediated actin polymerization at the ER stimulates mitochondrial calcium uptake, inner membrane constriction, and division. J. Cell Biol..

[112] Korobova F., Ramabhadran V., Higgs H.N. (2013). An actin-dependent step in mitochondrial fission mediated by the ER-associated formin INF2. Science.

[113] Wong Y.C., Ysselstein D., Krainc D. (2018). Mitochondria-lysosome contacts regulate mitochondrial fission via RAB7 GTP hydrolysis. Nature.

[114] Loson O.C., Song Z., Chen H., Chan D.C. (2013). Fis1, Mff, MiD49, and MiD51 mediate Drp1 recruitment in mitochondrial fission. Mol. Biol. Cell.

[115] Macdonald P., Stepanyants N., Singh A., Clinton R., Osellame L., Ryan M., Ramachandran R. (2018). MiD51 and Mff Co-assemble in Cardiolipin-Enriched Membrane Microdomains to Cooperatively Regulate Drp1-Mediated Mitochondrial Fission. Biophys. J..

[116] Francy C.A., Clinton R.W., Frohlich C., Murphy C., Mears J.A. (2017). Cryo-EM Studies of Drp1 Reveal Cardiolipin Interactions that Activate the Helical Oligomer. Sci. Rep..

[117] Nagashima S., Tabara L.C., Tilokani L., Paupe V., Anand H., Pogson J.H., Zunino R., McBride H.M., Prudent J. (2020). Golgi-derived PI(4)P-containing vesicles drive late steps of mitochondrial division. Science.

[118] Lee J.E., Westrate L.M., Wu H., Page C., Voeltz G.K. (2016). Multiple dynamin family members collaborate to drive mitochondrial division. Nature.

[119] Yu R., Jin S.B., Lendahl U., Nister M., Zhao J. (2019). Human Fis1 regulates mitochondrial dynamics through inhibition of the fusion machinery. EMBO J..

[120] Murakawa T., Yamaguchi O., Hashimoto A., Hikoso S., Takeda T., Oka T., Yasui H., Ueda H., Akazawa Y., Nakayama H. (2015). Bcl-2-like protein 13 is a mammalian Atg32 homologue that mediates mitophagy and mitochondrial fragmentation. Nat. Commun..

[121] Gustafsson A.B., Gottlieb R.A. (2008). Heart mitochondria: Gates of life and death. Cardiovasc. Res..

[122] Papanicolaou K.N., Ngoh G.A., Dabkowski E.R., O’Connell K.A., Ribeiro R.F., Stanley W.C., Walsh K. (2012). Cardiomyocyte deletion of mitofusin-1 leads to mitochondrial fragmentation and improves tolerance to ROS-induced mitochondrial dysfunction and cell death. Am. J. Physiol. Heart Circ. Physiol..

[123] Kalkhoran S.B., Hall A.R., White I.J., Cooper J., Fan Q., Ong S.B., Hernandez-Resendiz S., Cabrera-Fuentes H., Chinda K., Chakraborty B. (2017). Assessing the effects of mitofusin 2 deficiency in the adult heart using 3D electron tomography. Physiol. Rep..

[124] Papanicolaou K.N., Khairallah R.J., Ngoh G.A., Chikando A., Luptak I., O’Shea K.M., Riley D.D., Lugus J.J., Colucci W.S., Lederer W.J. (2011). Mitofusin-2 maintains mitochondrial structure and contributes to stress-induced permeability transition in cardiac myocytes. Mol. Cell Biol..

[125] Dorn G.W., Song M., Walsh K. (2015). Functional implications of mitofusin 2-mediated mitochondrial-SR tethering. J. Mol. Cell. Cardiol..

[126] Seidlmayer L.K., Mages C., Berbner A., Eder-Negrin P., Arias-Loza P.A., Kaspar M., Song M., Dorn G.W., Kohlhaas M., Frantz S. (2019). Mitofusin 2 Is Essential for IP3-Mediated SR/Mitochondria Metabolic Feedback in Ventricular Myocytes. Front. Physiol..

[127] Hall A.R., Burke N., Dongworth R.K., Kalkhoran S.B., Dyson A., Vicencio J.M., Dorn G.W., Yellon D.M., Hausenloy D.J. (2016). Hearts deficient in both Mfn1 and Mfn2 are protected against acute myocardial infarction. Cell Death Dis..

[128] Burke N., Hall A.R., Hausenloy D.J. (2015). OPA1 in Cardiovascular Health and Disease. Curr. Drug Targets.

[129] Piquereau J., Caffin F., Novotova M., Prola A., Garnier A., Mateo P., Fortin D., Huynh L.H., Nicolas V., Alavi M.V. (2012). Down-regulation of OPA1 alters mouse mitochondrial morphology, PTP function, and cardiac adaptation to pressure overload. Cardiovasc. Res..

[130] Le Page S., Niro M., Fauconnier J., Cellier L., Tamareille S., Gharib A., Chevrollier A., Loufrani L., Grenier C., Kamel R. (2016). Increase in Cardiac Ischemia-Reperfusion Injuries in Opa1^+/−^ Mouse Model. PLoS ONE.

[131] Bouche L., Kamel R., Tamareille S., Garcia G., Villedieu C., Pillot B., Gueguen N., Chehaitly A., Chao de la Barca J.M., Beaumont J. (2021). DRP1 haploinsufficiency attenuates cardiac ischemia/reperfusion injuries. PLoS ONE.

[132] Ikeda Y., Shirakabe A., Maejima Y., Zhai P., Sciarretta S., Toli J., Nomura M., Mihara K., Egashira K., Ohishi M. (2015). Endogenous Drp1 mediates mitochondrial autophagy and protects the heart against energy stress. Circ. Res..

[133] Ishihara T., Ban-Ishihara R., Maeda M., Matsunaga Y., Ichimura A., Kyogoku S., Aoki H., Katada S., Nakada K., Nomura M. (2015). Dynamics of mitochondrial DNA nucleoids regulated by mitochondrial fission is essential for maintenance of homogeneously active mitochondria during neonatal heart development. Mol. Cell Biol..

[134] Satohisa S., Zhang H.H., Feng L., Yang Y.Y., Huang L., Chen D.B. (2014). Endogenous NO upon estradiol-17beta stimulation and NO donor differentially regulate mitochondrial S-nitrosylation in endothelial cells. Endocrinology.

[135] Junior R.F.R., Rodrigues P.L., Morra E.A., Ronconi K.S., Do Val Lima P.R., Porto M.L., Simoes M.R., Vassallo D.V., Figueiredo S.G., Stefanon I. (2017). Estrogen regulates spatially distinct cardiac mitochondrial subpopulations. Mitochondrion.

[136] Martin O.J., Lai L., Soundarapandian M.M., Leone T.C., Zorzano A., Keller M.P., Attie A.D., Muoio D.M., Kelly D.P. (2014). A role for peroxisome proliferator-activated receptor gamma coactivator-1 in the control of mitochondrial dynamics during postnatal cardiac growth. Circ. Res..

[137] Dworatzek E., Mahmoodzadeh S., Schubert C., Westphal C., Leber J., Kusch A., Kararigas G., Fliegner D., Moulin M., Ventura-Clapier R. (2014). Sex differences in exercise-induced physiological myocardial hypertrophy are modulated by oestrogen receptor beta. Cardiovasc. Res..

[138] Du M., Shan J., Feng A., Schmull S., Gu J., Xue S. (2020). Oestrogen Receptor beta Activation Protects Against Myocardial Infarction via Notch1 Signalling. Cardiovasc. Drugs Ther..

[139] Ong S.B., Hall A.R., Dongworth R.K., Kalkhoran S., Pyakurel A., Scorrano L., Hausenloy D.J. (2015). Akt protects the heart against ischaemia-reperfusion injury by modulating mitochondrial morphology. Thromb. Haemost..

[140] Lagranha C.J., Deschamps A., Aponte A., Steenbergen C., Murphy E. (2010). Sex differences in the phosphorylation of mitochondrial proteins result in reduced production of reactive oxygen species and cardioprotection in females. Circ. Res..

[141] Paris O., Ferraro L., Grober O.M., Ravo M., De Filippo M.R., Giurato G., Nassa G., Tarallo R., Cantarella C., Rizzo F. (2012). Direct regulation of microRNA biogenesis and expression by estrogen receptor beta in hormone-responsive breast cancer. Oncogene.

[142] Wang K., Liu C.Y., Zhang X.J., Feng C., Zhou L.Y., Zhao Y., Li P.F. (2015). miR-361-regulated prohibitin inhibits mitochondrial fission and apoptosis and protects heart from ischemia injury. Cell Death Differ..

[143] Amput P., Palee S., Arunsak B., Pratchayasakul W., Thonusin C., Kerdphoo S., Jaiwongkam T., Chattipakorn S.C., Chattipakorn N. (2020). PCSK9 inhibitor and atorvastatin reduce cardiac impairment in ovariectomized prediabetic rats via improved mitochondrial function and Ca(2+) regulation. J. Cell. Mol. Med..

[144] Souvenir R.A., Renata A., Campbell R., Hinton A.O., Rondina M.T., Abel E.D. (2020). SUN-572 Estrogen Synergistically Interacts with Optic Atrophy Protein 1 to Promote Thrombosis. J. Endocr. Soc..

[145] Devine M.J., Kittler J.T. (2018). Mitochondria at the neuronal presynapse in health and disease. Nat. Rev. Neurosci..

[146] Hollenbeck P.J., Saxton W.M. (2005). The axonal transport of mitochondria. J. Cell Sci..

[147] Li Z., Okamoto K., Hayashi Y., Sheng M. (2004). The importance of dendritic mitochondria in the morphogenesis and plasticity of spines and synapses. Cell.

[148] Bevan R.J., Williams P.A., Waters C.T., Thirgood R., Mui A., Seto S., Good M., Morgan J.E., Votruba M., Erchova I. (2020). OPA1 deficiency accelerates hippocampal synaptic remodelling and age-related deficits in learning and memory. Brain Commun..

[149] Smith T.G., Seto S., Ganne P., Votruba M. (2016). A randomized, placebo-controlled trial of the benzoquinone idebenone in a mouse model of OPA1-related dominant optic atrophy reveals a limited therapeutic effect on retinal ganglion cell dendropathy and visual function. Neuroscience.

[150] Trevisan T., Pendin D., Montagna A., Bova S., Ghelli A.M., Daga A. (2018). Manipulation of Mitochondria Dynamics Reveals Separate Roles for Form and Function in Mitochondria Distribution. Cell Rep..

[151] Ishihara N., Nomura M., Jofuku A., Kato H., Suzuki S.O., Masuda K., Otera H., Nakanishi Y., Nonaka I., Goto Y. (2009). Mitochondrial fission factor Drp1 is essential for embryonic development and synapse formation in mice. Nat. Cell Biol..

[152] Verstreken P., Ly C.V., Venken K.J., Koh T.W., Zhou Y., Bellen H.J. (2005). Synaptic mitochondria are critical for mobilization of reserve pool vesicles at Drosophila neuromuscular junctions. Neuron.

[153] Kageyama Y., Zhang Z., Roda R., Fukaya M., Wakabayashi J., Wakabayashi N., Kensler T.W., Reddy P.H., Iijima M., Sesaki H. (2012). Mitochondrial division ensures the survival of postmitotic neurons by suppressing oxidative damage. J. Cell Biol..

[154] Chen H., McCaffery J.M., Chan D.C. (2007). Mitochondrial fusion protects against neurodegeneration in the cerebellum. Cell.

[155] Han S., Nandy P., Austria Q., Siedlak S.L., Torres S., Fujioka H., Wang W., Zhu X. (2020). Mfn2 Ablation in the Adult Mouse Hippocampus and Cortex Causes Neuronal Death. Cells.

[156] Zhou Y., Carmona S., Muhammad A., Bell S., Landeros J., Vazquez M., Ho R., Franco A., Lu B., Dorn G.W. (2019). Restoring mitofusin balance prevents axonal degeneration in a Charcot-Marie-Tooth type 2A model. J. Clin. Investig..

[157] Lee S., Sterky F.H., Mourier A., Terzioglu M., Cullheim S., Olson L., Larsson N.G. (2012). Mitofusin 2 is necessary for striatal axonal projections of midbrain dopamine neurons. Hum. Mol. Genet..

[158] Hara Y., Yuk F., Puri R., Janssen W.G., Rapp P.R., Morrison J.H. (2014). Presynaptic mitochondrial morphology in monkey prefrontal cortex correlates with working memory and is improved with estrogen treatment. Proc. Natl. Acad. Sci. USA.

[159] Arnold S., de Araujo G.W., Beyer C. (2008). Gender-specific regulation of mitochondrial fusion and fission gene transcription and viability of cortical astrocytes by steroid hormones. J. Mol. Endocrinol..

[160] Sung J.H., Cho E.H., Min W., Kim M.J., Kim M.O., Jung E.J., Koh P.O. (2010). Identification of proteins regulated by estradiol in focal cerebral ischemic injury--a proteomics approach. Neurosci. Lett..

[161] Demarest T.G., Waite E.L., Kristian T., Puche A.C., Waddell J., McKenna M.C., Fiskum G. (2016). Sex-dependent mitophagy and neuronal death following rat neonatal hypoxia-ischemia. Neuroscience.

[162] Namjoo Z., Moradi F., Aryanpour R., Piryaei A., Joghataei M.T., Abbasi Y., Hosseini A., Hassanzadeh S., Taklimie F.R., Beyer C. (2018). Combined effects of rat Schwann cells and 17beta-estradiol in a spinal cord injury model. Metab. Brain Dis..

[163] Kumari S., Mehta S.L., Milledge G.Z., Huang X., Li H., Li P.A. (2016). Ubisol-Q10 Prevents Glutamate-Induced Cell Death by Blocking Mitochondrial Fragmentation and Permeability Transition Pore Opening. Int. J. Biol. Sci..

[164] Koh P.O. (2007). 17Beta-estradiol prevents the glutamate-induced decrease of Akt and its downstream targets in HT22 cells. J. Vet. Med. Sci..

[165] Giordano C., Montopoli M., Perli E., Orlandi M., Fantin M., Ross-Cisneros F.N., Caparrotta L., Martinuzzi A., Ragazzi E., Ghelli A. (2011). Oestrogens ameliorate mitochondrial dysfunction in Leber’s hereditary optic neuropathy. Brain.

[166] Sarkar S., Jun S., Simpkins J.W. (2015). Estrogen amelioration of Abeta-induced defects in mitochondria is mediated by mitochondrial signaling pathway involving ERbeta, AKAP and Drp1. Brain Res..

[167] Prat A., Behrendt M., Marcinkiewicz E., Boridy S., Sairam R.M., Seidah N.G., Maysinger D. (2011). A novel mouse model of Alzheimer’s disease with chronic estrogen deficiency leads to glial cell activation and hypertrophy. J. Aging Res..

[168] Yao J., Irwin R., Chen S., Hamilton R., Cadenas E., Brinton R.D. (2012). Ovarian hormone loss induces bioenergetic deficits and mitochondrial beta-amyloid. Neurobiol. Aging.

[169] Duarte A.I., Candeias E., Alves I.N., Mena D., Silva D.F., Machado N.J., Campos E.J., Santos M.S., Oliveira C.R., Moreira P.I. (2020). Liraglutide Protects Against Brain Amyloid-beta1-42 Accumulation in Female Mice with Early Alzheimer’s Disease-Like Pathology by Partially Rescuing Oxidative/Nitrosative Stress and Inflammation. Int. J. Mol. Sci..

[170] Tahsili-Fahadan P., Geocadin R.G. (2017). Heart-Brain Axis: Effects of Neurologic Injury on Cardiovascular Function. Circ. Res..

[171] Riching A.S., Major J.L., Londono P., Bagchi R.A. (2020). The Brain-Heart Axis: Alzheimer’s, Diabetes, and Hypertension. ACS Pharmacol. Transl. Sci..

[172] Kerro A., Woods T., Chang J.J. (2017). Neurogenic stunned myocardium in subarachnoid hemorrhage. J. Crit. Care.

[173] Ashby E.L., Miners J.S., Kehoe P.G., Love S. (2016). Effects of Hypertension and Anti-Hypertensive Treatment on Amyloid-beta (Abeta) Plaque Load and Abeta-Synthesizing and Abeta-Degrading Enzymes in Frontal Cortex. J. Alzheimers Dis..

[174] Sundboll J., Horvath-Puho E., Adelborg K., Schmidt M., Pedersen L., Botker H.E., Henderson V.W., Sorensen H.T. (2018). Higher Risk of Vascular Dementia in Myocardial Infarction Survivors. Circulation.

[175] Arboix A. (2015). Cardiovascular risk factors for acute stroke: Risk profiles in the different subtypes of ischemic stroke. World J. Clin. Cases.

[176] Mrozek S., Gobin J., Constantin J.M., Fourcade O., Geeraerts T. (2020). Crosstalk between brain, lung and heart in critical care. Anaesth. Crit. Care Pain Med..

[177] Gao J., Xu D., Sabat G., Valdivia H., Xu W., Shi N.Q. (2014). Disrupting KATP channels diminishes the estrogen-mediated protection in female mutant mice during ischemia-reperfusion. Clin. Proteom..

[178] Minta W., Palee S., Mantor D., Sutham W., Jaiwongkam T., Kerdphoo S., Pratchayasakul W., Kumfu S., Chattipakorn S.C., Chattipakorn N. (2018). Estrogen deprivation aggravates cardiometabolic dysfunction in obese-insulin resistant rats through the impairment of cardiac mitochondrial dynamics. Exp. Gerontol..

[179] Carnevale D., Mascio G., D’Andrea I., Fardella V., Bell R.D., Branchi I., Pallante F., Zlokovic B., Yan S.S., Lembo G. (2012). Hypertension induces brain beta-amyloid accumulation, cognitive impairment, and memory deterioration through activation of receptor for advanced glycation end products in brain vasculature. Hypertension.

[180] Ribaric S. (2016). The Rationale for Insulin Therapy in Alzheimer’s Disease. Molecules.

[181] Previtali M., Repetto A., Panigada S., Camporotondo R., Tavazzi L. (2009). Left ventricular apical ballooning syndrome: Prevalence, clinical characteristics and pathogenetic mechanisms in a European population. Int. J. Cardiol..

[182] Templin C., Ghadri J.R., Diekmann J., Napp L.C., Bataiosu D.R., Jaguszewski M., Cammann V.L., Sarcon A., Geyer V., Neumann C.A. (2015). Clinical Features and Outcomes of Takotsubo (Stress) Cardiomyopathy. N. Engl. J. Med..

[183] El-Battrawy I., Zhao Z., Lan H., Schunemann J.D., Sattler K., Buljubasic F., Patocskai B., Li X., Yucel G., Lang S. (2018). Estradiol protection against toxic effects of catecholamine on electrical properties in human-induced pluripotent stem cell derived cardiomyocytes. Int. J. Cardiol..

[184] Xu S., Wang P., Zhang H., Gong G., Gutierrez Cortes N., Zhu W., Yoon Y., Tian R., Wang W. (2016). CaMKII induces permeability transition through Drp1 phosphorylation during chronic beta-AR stimulation. Nat. Commun..

